# Tackling Smoker Misperceptions About E-cigarettes Using Expert Videos

**DOI:** 10.1093/ntr/ntab104

**Published:** 2021-05-20

**Authors:** Madeleine Svenson, James Green, Olivia M Maynard

**Affiliations:** 1 Department of Psychology, University of Bath, Bath, UK; 2 School of Psychological Science and MRC Integrative Epidemiology Unit (IEU), University of Bristol, Bristol, UK

## Abstract

**Background:**

The pervasive misperception that e-cigarettes are equally or more harmful than combustible cigarettes is a barrier to current smokers switching to e-cigarettes. To tackle misperceptions, public health bodies are using informational videos, although their efficacy is unknown.

**Methods:**

In our online study, current UK smokers who do not vape (*n* = 382) were randomized to view either: (1) a Cancer Research UK (CRUK) text-only video; (2) a video featuring leading e-cigarette experts (expert); or (3) a no video control condition, and then completed questions regarding e-cigarette harm perceptions.

**Results:**

Compared to the control condition, participants in the CRUK condition, and especially those in the expert condition had more accurate harm perceptions of e-cigarettes and had more accurate knowledge of e-cigarette constituents. In the expert condition, 67% of individuals reported they would try an e-cigarette in a future quit attempt, compared with 51% in the CRUK condition and 35% in the control condition.

**Conclusions:**

Our findings are encouraging in the face of mounting evidence that e-cigarette misperceptions are increasing. Whilst misperceptions are often characterized as resistant to correction, we find that carefully designed public health information videos have the potential to promote a more accurate, informed view of e-cigarettes, and encourage intended e-cigarette use among UK smokers. Importantly, we find this among current smokers who do not vape, a group often reported as having the highest levels of misperceptions and as having the most to gain from accurate e-cigarette perceptions.

**Implications:**

There is mounting evidence that e-cigarette misperceptions are increasing, particularly among smokers who do not vape, a group who have most to gain from accurate information about e-cigarettes. Misperceptions are often characterized as difficult to change and there is relatively little research on how to correct e-cigarette misperceptions. Our research in the UK shows that, compared to controls, e-cigarette misperceptions can be corrected among those smokers who are shown carefully constructed expert videos. This work has important implications for the development and dissemination of these important messages.

## Introduction

E-cigarettes represent an opportunity to stop tobacco use for many smokers. They are estimated to carry less than 5% of the harm of combustible cigarettes^1^ and recent evidence shows that they can support people to stop smoking.^2^ E-cigarettes, therefore, represent both an effective smoking cessation technique and a significantly reduced harm alternative to smoking.

Despite this, e-cigarettes are still met with widespread uncertainty by smokers. A Public Health England report estimates that 40% of smokers have never tried an e-cigarette, often due to misperceptions surrounding its safety.^2^ Over time, these misperceptions appear to be becoming more prominent. Between 2014 and 2019, the proportion of English smokers who correctly believed e-cigarettes were less harmful than cigarettes reduced from 45% to 34%.^3^ This pattern is mirrored in the US^4^ and inaccurate harm perceptions are most common among smokers who do not vape.^3^ There is, therefore, an important public health need to challenge these widespread misperceptions.

Care must be taken when designing and disseminating corrective information. A meta-analysis of the misinformation literature finds that corrective information is rarely fully effective and misinformation often persists, even after debunking attempts.^5^ Indeed, misinformation still influences decisions after corrections are acknowledged, and more concerningly, corrective information often backfires, accidently reinforcing the misperceptions it is designed to challenge.^6–10^ Given these complexities, corrective information must be carefully constructed, and its efficacy established, ideally before public dissemination. This is especially important to avoid any backfire effects that would render informational campaign counterproductive.

There have been numerous public health campaigns designed to better educate the public and challenge misperceptions surrounding e-cigarettes, and there is some indication that these are effective. Among US adult smokers, self-reported exposure to accurate e-cigarette information was associated with lower e-cigarette harm perceptions^11^ and experimental exposure to pictorial messages comparing e-cigarettes with cigarettes reduces e-cigarette risk perceptions.^12^ Perceived source credibility has also been shown to be related to more positive e-cigarette attitudes.^13^ Indeed, expert consensus and source credibility are known to be useful tools for correcting misperceptions.^14,15^

There is a need to test the efficacy of corrective public health information to ensure that they are having a measurable effect in the desired direction. Here we examine the impact of informational videos from trusted, expert sources on e-cigarette harm perceptions among current smokers who do not vape. From a public health perspective, it is most important to study the impact of these videos among this group, as their health would benefit the most from switching to e-cigarettes and they report the highest endorsement of e-cigarette misperceptions.^3^

We hypothesized that participants who viewed information campaigns from trusted sources (either Cancer Research UK or a panel of scientific experts) would have fewer misperceptions about e-cigarettes than those who do not view any campaigns. Moreover, a video campaign with leading experts will be more effective at reducing misperceptions than the text-only animated video campaign by CRUK. This study differs from the majority of previous work in this field, which typically uses a single item measure of perceived harm.^16^ Here we use multiple items, as has been recommended, to increase the validity of this measure.^17^ This also allows us to (1) identify the specific misbeliefs that underpin negative perceptions towards e-cigarettes in smokers, so that these can be individually targeted in future public information campaigns, and (2) explore the impact of the informational campaigns on specific harm perception beliefs, giving us a more detailed picture of the efficacy of pre-existing informational campaigns.

## Method

### Design and Overview

We conducted an online between-subjects experimental study in February 2020 with UK smokers who do not use e-cigarettes (i.e., do not vape). Participants were randomized into one of the three conditions: CRUK video; expert video; or a no message control. Harm perceptions were the primary outcome measure. We published the study protocol on the Open Science Framework prior to starting testing and this includes more information about the study methods and the analysis plan (https://osf.io/ja34v/).

### Participants

Our pre-planned sample size calculation indicated that we needed 390 participants to observe a small effect size (*f* = 0.20) with 95% power and an alpha level of 5%. Further details are in the pre-registered protocol. We recruited an equal number of female and male participants opportunistically through Prolific, an online crowdsourcing platform. To be eligible for inclusion, participants were at least 18 years of age, lived in the UK and self-reported that they smoked daily, and did not vape (classified as using an e-cigarette less than monthly). Participants were reimbursed 13p for completing a short prescreening survey. Eligible participants were invited to participate in the main, 10 min experiment on Qualtrics for which they were reimbursed £1.30. The study was approved by the Faculty of Science Research Ethics Committee at the University of Bristol (reference: 23051753685).

### Materials and Measures

The pre-registered protocol provides a detailed description of all measures (https://osf.io/ja34v/).

#### Experimental Stimuli – E-cigarette Videos

We used existing video information campaigns as stimuli in the CRUK and expert conditions. Participants randomly assigned to the CRUK condition saw a video published by CRUK (video stills in the Supplementary Materials and viewable at https://www.youtube.com/watch?v=9BEdv7UTDBA&feature=emb_logo). CRUK is the world’s largest independent cancer charity, fundraising over half a billion in the financial year ending March 2019.^18,19^ In 2013, CRUK was voted the most trusted charity in the UK.^20^ The CRUK video is a text based animated video lasting 30 s. It included CRUK’s logo and simple pictograms, alongside the following statements: (1) Research shows vaping is far less harmful than smoking; (2) E-cigarettes contain nicotine, which is addictive, but does not cause cancer; (3) E-cigarettes do not contain cancer-causing tobacco; 4) Passively breathing vapor from e-cigarettes is unlikely to be harmful; and 5) Growing evidence shows e-cigarettes are helping people to stop smoking.

Participants who were randomly assigned to the expert condition viewed a video (2 min 20 s) published by the National Centre for Smoking Cessation and Training (NCSCT) in association with the New Nicotine Alliance and part funded by Public Health England (viewable at https://www.youtube.com/watch?v=SSn5ZZQkzKs or alternatively see the Supplementary Materials for a transcript and video stills). The video features four leading experts who address common e-cigarette misperceptions. These include: the contents of e-cigarette vapor and combustible cigarette smoke; e-cigarette use as a smoking cessation technique; the “gateway” theory and the relative safety of vaping compared to smoking combustible cigarettes.

#### Perceptions of E-cigarettes

We asked what we call “general harm-reduction statements”: (1) I know enough about e-cigarettes to have formed accurate opinions; (2) e-cigarettes are harmful; (3) e-cigarettes are less harmful than combustible cigarettes; (4) e-cigarettes are a helpful tool for people who want to quit smoking; (5) there is convincing scientific evidence that e-cigarettes are safe; (6) there is convincing scientific evidence that e-cigarettes are safer than smoking. We also asked what we called “specific harm-reduction statements”: (7) the health risks of smoking come from tar in combustible cigarettes; (8) the health risks of smoking come from nicotine in combustible cigarettes; (9) e-cigarettes often contain tar; (10) e-cigarettes often contain chemicals that are harmful to the user’s health; (11) there is a high risk of harmful accidents when using e-cigarettes; (12) secondhand e-cigarette vapor can expose others to harm; and (13) e-cigarettes normalize smoking, making more young people take up smoking. Participants reported e-cigarette harm perceptions using a 7-point scale, with 1 representing “strongly agree” and 7 representing “strongly disagree,” with an additional “don’t know” option. We note that for some of these items (e.g., items 2, 5, and 10) the “correct” answer according to the current science may not be at either of the extremes of our 7-point scale (but will be close to the extremes), given that e-cigarettes are estimated to be 95% (but not 100%) less harmful than cigarettes.^21^

We also asked participants for their biggest concern about e-cigarettes, with the options “their harmful contents,” “their addictiveness,” “the potential for freak accidents,” “their harm to others,” “they are normalizing smoking for young people”, and “other” with an option for them to provide details.

#### Smoking and Vaping Behavior

Participants were asked about their frequency of cigarette and e-cigarette use, the number of cigarettes smoked per day, and the number of serious smoking quit attempts they had made. Participants also completed the Quitting Smoking Contemplation Ladder^22^ and the Fagerström Test of Cigarette Dependence.^23^

To assess intentions to use an e-cigarette, participants were asked: “Do you think that you will try an e-cigarette or vaping device soon?” and “My future quit attempts will involve an e-cigarette or vaping device,” with the options “definitely not,” “probably not,” “probably yes”, and “definitely yes.”

#### Demographics

Participants reported their age, location in the UK, gender, level of education, university student status, ethnicity, and type of occupation.

#### Attention Checks

Immediately after watching the video (or after consent for the control condition), participants were asked “From who did you just watch a video” with the options (1) Cancer Research UK, (2) British Heart Foundation, (3) A panel of experts, and (4) I did not watch a video, with the correct answer dependent on their condition.

An additional attention check item was presented at random within the attitudes to vaping questions, where participants were asked to “respond with Strongly Agree.”

### Procedure

After providing informed consent, participants provided their Prolific ID, and then were randomized (via Qualtrics) to one of the three conditions. Participants were unaware of the presence or indeed content of the other conditions. Immediately prior to watching the videos, participants were instructed to ensure they were in a location they could listen to and watch the videos, and were informed that they must pay close attention as they would be asked questions about them afterwards. Participants in the control condition were asked to tick a box saying that they would answer the following questions carefully.

Participants then completed the video attention check and then completed the questions on perceptions of e-cigarettes, smoking and vaping behavior, and demographics. Participants then had an opportunity to provide any comments or questions in a free-text box and were then debriefed (with all participants provided with links to the two videos) and redirected to Prolific for reimbursement.

### Statistical Analysis Plan

For the harm perceptions statements, a mean was taken of each statement, with “Don’t know” responses removed. Shapiro–Wilk’s test confirmed that means for each statement were non-normally distributed and Levene’s test indicated unequal variance for 8 of 13 statements. Despite these findings, we report the results of planned one-way ANOVAs on these outcomes, as ANOVA is relatively robust to violations of normality^24^ and the results of the Brown–Forsythe tests mirrored the ANOVA results. We used the Games-Howell procedure for post hoc tests due to the unequal variance.

For the question regarding participants’ biggest concern about e-cigarettes, responses were counted to reveal the greatest concerns and we supplemented this with an assessment of the free text responses. For behavioral intentions related to using an e-cigarette in the future, we used chi-squared analyses to examine the difference in proportion of participants in each condition who reported that they either “probably” or “definitely” would use an e-cigarette in the future and would use one in a future quit attempt.

All questions were “forced choice” so there were no missing data. We removed from the analyses any participants who failed either of the attention check questions (*n* = 25). Our pre-registered statistical analysis plan (see https://osf.io/ja34v/) stated that we would run analyses with and without those who failed the attention check questions. However, given the small number of exclusions and for simplicity in reporting, we have not performed or reported this analysis. We used SPSS (version 25) for the quantitative data analysis and the researchers involved were not blinded to the study conditions while performing the analyses. We have avoided using the term “significant” or “nonsignificant,” given the binary nature of the threshold that these terms rely upon.^25^ Instead, we use terms such as “weak” and “strong evidence” to reflect the strength of the evidence, and we determine this using a range of factors including the effect size estimates, exact *p* values, the direction of the point estimate and whether that is consistent with our a priori predictions.

## Results

The data and analysis code that form the basis of the results presented here are available from the University of Bristol data repository, data.bris, at https://data.bris.ac.uk/data/dataset/8j9tx4rrrfu822xmuluahaunb; DOI: 10.5523/bris.8j9tx4rrrfu822xmuluahaunb.

### Characteristics of Participants

Detailed participant characteristics are shown in Table 1 and in Supplementary Table 1. There were 433 participants who completed the experiment, 26 were excluded for not being daily smokers and less than monthly vapers, and 25 were excluded for failing either of the attention checks. This left a total of 382 participants (control *n* = 132, CRUK, *n* = 121, expert, *n* = 129).

**Table 1.** Participant Characteristics

Participant characteristic	Participants (*n* = 382)	Control (*n* = 132)	CRUK (*n* = 121)	Expert (*n* = 129)
Gender				
Female	200 (52%)	61 (46%)	64 (53%)	75 (58%)
Male	182 (48%)	71 (54%)	57 (47%)	54 (42%)
Age	38.5 (SD ± 11.8)	40.3 (SD ± 12.0)	37.9 (SD ± 11.3)	37.3 (SD ± 11.8)
Minimum cigarettes smoked per day last 2 months	9.0 (SD ± 16.2)	10.8 (SD ± 26.1)	8.2 (SD ± 6.7)	8.0 (SD ± 5.8)
Maximum cigarettes smoked per day last 2 months	29.6 (SD ± 153.7)	25.0 (SD ± 31.5)	43.5 (SD ± 271.1)	21.2 (SD ± 10.0)
Number of previous quit attempts	2.6 (SD ± 3.7)	2.6 (SD ± 3.2)	2.3 (SD ± 1.7)	2.9 (SD ± 5.2)
Nicotine dependence (FTCD)	4.5 (SD ± 1.0)	4.4 (SD ± 1.0)	4.5 (SD ± 1.0)	4.5 (SD ± 1.0)
Vaping status				
Vape less than monthly	80 (21%)	21 (16%)	28 (23%)	31 (24%)
Never vaped/vape less than monthly	302 (79%)	111 (84%)	93 (77%)	98 (76%)

FCND, Fagerström Test of Cigarette Dependence.

### E-cigarette Harm Perceptions

**Figure 1.**
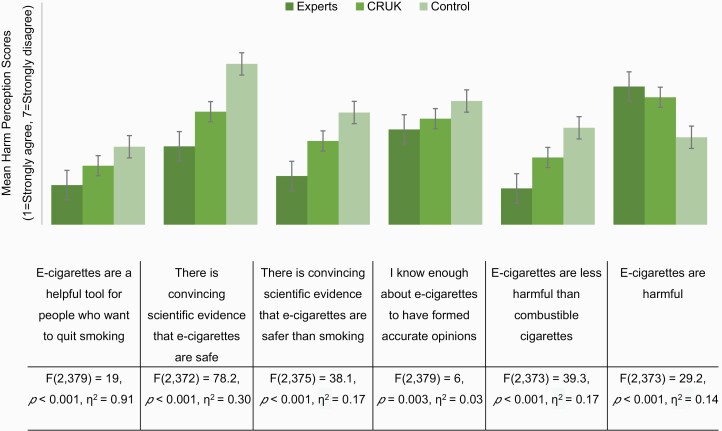
Mean response to general harm perception statements on a 7-point scale, split by video condition. 1 represents strongly agree and 7 represents strongly disagree. Therefore, higher means indicate higher disagreement with the harm perception statement. Error bars represent ±1 SE. “Do not know” responses were coded as missing. For each harm perception statement, the results of a one-way ANOVA with eta squared are displayed.

**Figure 2.**
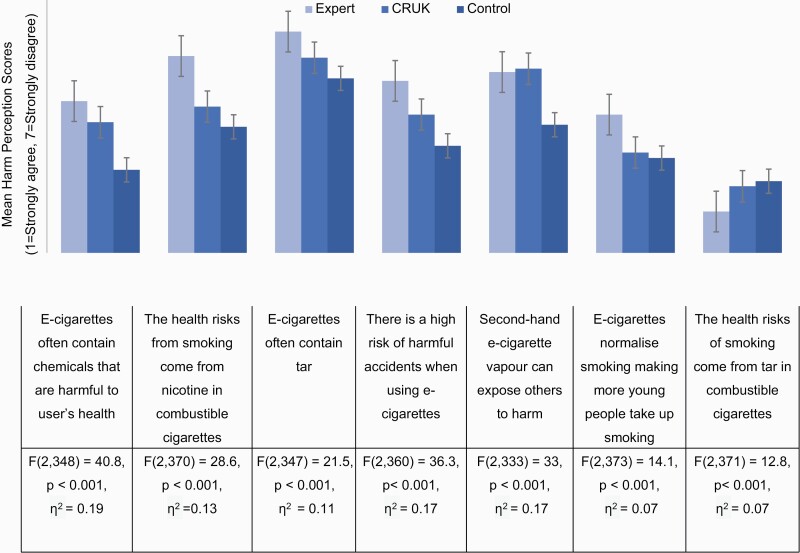
Mean response to specific harm perception statements on a 7-point scale, split by video condition. 1 represents strongly agree and 7 represents strongly disagree. Therefore, higher means indicate higher disagreement with the harm perception statement. Error bars represent ±1 SE. “Do not know” responses were coded as missing. For each harm perception statement, the results of a oneway ANOVA with eta squared are displayed.

There was a main effect of experimental condition on each of the 13 harm-reduction statements (see Figures 1 and 2 for results including test statistics). Differences between conditions were in the expected direction—that is, we observed the lowest e-cigarette harm perceptions among those in the expert condition, followed by the CRUK condition, with those in the control condition with the highest e-cigarette harm perceptions. Post hoc comparisons (Supplementary Table 2) indicated that there were large differences between the expert and control conditions for all harm perception statements. There were also large differences in the expected direction between the CRUK and control, and between expert and CRUK conditions for most harm perception statements.

### Biggest Concerns About E-cigarettes

Table 2 presents participants’ biggest concerns about e-cigarettes. A smaller percentage of those in the expert condition (21%) reported that their biggest concern was the harmful contents of e-cigarettes, compared to 36% in the CRUK and 44% in the control condition.

**Table 2.** Biggest Concern about E-cigarettes

Biggest concern about e-cigarettes	Overall (*n* = 382)	Control (*n* = 132)	CRUK (*n* = 121)	Expert (*n* = 129)
	*n* (%)	*n* (%)	*n* (%)	*n* (%)
Their harmful contents	129 (34)	58 (44)	44 (36)	27 (21)
The potential for freak accidents	67 (18)	15 (12)	18 (15)	34 (26)
Their addictiveness	66 (17)	15 (12)	26 (22)	25 (19)
They are normalising smoking for young people	47 (12)	20 (15)	13 (11)	14 (11)
Their harm to others	1 (0.3)	1 (0.8)	0 (0)	0 (0)
Other	72 (19)	23 (17)	20 (17)	29 (23)

Of the 72 individuals reporting “other” as their main concern, 64 provided further qualitative information. Coding of these responses suggested the primary concern was epistemic limitations. Of the 28 participants expressing these concerns about the state of knowledge, 12 specifically expressed concern about the long-term safety of e-cigarettes being unknown. The second most reported “other” concern (*n* = 8) was the notion that e-cigarettes do not promote nicotine abstinence and may encourage dual use. Another commonly reported concern was that e-cigarettes were an inadequate replacement, which did not provide the same satiety or enjoyment as cigarettes.

### Behavioral Intentions

The videos also impacted participants’ behavioral intentions regarding e-cigarettes. The chi-squared analysis indicated an association between condition and people reporting that they would try e-cigarettes in the future (*χ*^2^_(2, *N* = 382)_ = 32.5, *p* < .001, *V* = .21). This was predominantly driven by large differences in willingness to try e-cigarettes between the expert and control condition, with the highest percentage in the expert condition (59%), followed by those in the CRUK condition (43%), with the lowest percentage in the control condition (31%).

There was also a small association between condition and participants reporting they would use e-cigarettes in a future quit attempt (*χ*^2^_(2, *N* = 382)_ = 41.61, *p* < .001, *V* = .23), with the highest endorsement of this in the expert condition (67%), followed by the CRUK condition (51%), followed by the control condition (35%).

## Discussion

We find that exposure to a short CRUK infographic video, and especially a video featuring e-cigarette experts, meaningfully corrected e-cigarette misperceptions among adult smokers who do not vape and increased their intentions to try an e-cigarette, compared to control participants who saw no video. Supporting previous findings, we observed high levels of misperceptions among those in our control group who saw no video, with these participants providing stronger endorsement for misbeliefs such as “secondhand e-cigarette vapor can expose others to harm” and “e-cigarettes often contain chemicals that are harmful to users’ health”. Given the potential of e-cigarettes to reduce harm among current smokers, our findings indicate the significant public health potential of these brief video interventions.^26,27^ Previous work finds that perceiving e-cigarettes as less harmful than cigarettes predicts later e-cigarette use among never-vapers^28^ and this is supported by our finding that participants who watched either video expressed increased intentions to use e-cigarettes in the future compared with the control group.

Interestingly, we also found evidence of “spill-over effects,” such that individuals in the CRUK and expert condition displayed lower endorsement of the risk of accidents compared to the control, even though neither video directly addressed this harm. Similarly, those who watched the expert video, compared to control, were more likely to think that there was not a secondhand smoke risk from e-cigarettes, even though the video does not mention this. These findings suggest that the videos promote a generally more positive attitude towards e-cigarettes. These findings raise the question of how much information is actually required to promote these positive attitudes and spill-over effects. As we discuss below, keeping informational videos as short and focused as possible is likely to be the key to their success in reaching large audiences (e.g., on television and social media where content must be attention-grabbing and brief). Future work should explore which messages within these videos are most important in promoting positive attitudes towards vaping among current smokers and which are most likely to promote spill-over to other misperceptions.

However, not all of the information in the videos translated to changes in harm perceptions. The misbelief that the health risks of smoking are due to nicotine did not differ between the CRUK and control condition, although the CRUK video states “E-cigarettes contain nicotine, nicotine is addictive but does not cause cancer.” In contrast, the expert video states “People die from the tar, the other constituents of smoke, but not the nicotine. So the nicotine isn’t the harmful component” and in this condition we did see a large decrease in endorsement of the statement that nicotine in cigarettes causes health risks. The divergent results may be due to differences in the video format, such as expertise, or speaking versus text. Alternatively, the effect may be driven by the semantic differences between “addictive but does not cause cancer” and “isn’t the harmful component,” or perhaps by explicitly ascribing the harm to the tar. As our methodology does not identify which specific aspect of the video drives the positive changes to harm perceptions of e-cigarettes, this would be an interesting avenue for future research.

We also investigated smokers’ biggest concerns about e-cigarettes. Of the list we provided, concern about the constituents of e-cigarettes was common. However, many participants reported other concerns that were not on our list, notably the limits of our knowledge about e-cigarettes, including concerns about the long-term health consequences of e-cigarettes. Along with our detailed information about 13 different potential misperceptions, our research adds to the literature which characterizes the nature and extent of e-cigarette misperceptions among adult smokers.^29^ This should be used to inform future public health informational videos.

There is considerable concern about these misperceptions, as misinformation is often conceptualized as being resistant to correction. Misperceptions are often maintained and entrenched through motivated reasoning, with counter-attitudinal information typically dismissed.^5^ The effectiveness of correction is, therefore, determined in part by the receptivity of the target audience to it.^30^ Although there is now considerable evidence on the presence of e-cigarette misperceptions among smokers, relatively little research has examined how to correct these misperceptions.^3,17,28^ Our research not only finds that misperceptions can be corrected (without backfire effects), but that this can be achieved among nonvapers (79% reported never vaping), a group for whom e-cigarette misinformation is greatest^3^ and who have the most to gain from accurate e-cigarette knowledge—this is particularly encouraging and is a key strength of our work.

There are, however, some important limitations to our work. First, our sample is not representative of the UK, with White participants being over-represented, Second, we measured behavioral intentions, rather than actual vaping behaviors. Whilst intentions are informative, they do not always directly translate into behavior and future work could assess e-cigarette uptake one month later. Third, as we wanted to assess the impact of currently available e-cigarette videos, the content of these was not standardized. This means we are unable to make firm conclusions about which components of these videos (e.g., video length, the presence of experts, the content or the semantics of what was said or written etc) had the greatest influence on perceptions. Finally and importantly, our study uses a forced exposure paradigm which lacks ecological validity and, therefore, limits the conclusions we can draw about the effectiveness of these videos outside of our experiment. Health communication campaigns must reach their target audience to be effective^31^ and it is likely that under naturalistic conditions, the vast majority of our target sample of nonvaping smokers may not even become aware of the videos, let alone watch them in their entirety whilst paying close attention. Indeed, Festinger’s theory of cognitive dissonance suggests that counter-attitudinal information is aversive, so individuals may avoid videos such as these that challenge their beliefs and behaviors.^32,33^ This self-selection of media content is becoming increasingly common due to the proliferation of health information on the Internet.^34^ However, as of January 2021, the CRUK video has received 9855 views and the expert video 21 736 views. Given we find that both videos can reduce misperceptions when close attention is paid to them, consideration of how to increase engagement, particularly among target groups is important. This may include paying for these videos to appear as advertisements either online on video streaming platforms or on television. Key information could also be translated to written format (e.g., via infographics) to facilitate broad, multimedia dissemination.

To conclude, we find that compared to controls, short informational videos can reduce e-cigarette harm perceptions and increase intentions to use e-cigarettes among current smokers who do not vape. A video which uses experts to debunk common myths was particularly effective. Our results are encouraging in the face of mounting evidence that e-cigarette misperceptions are increasing. Whilst research finds that misinformation is often resistant to correction, we find that attitude change in this domain is possible, at least when we use carefully designed public health campaigns. Future research should consider which messages within these videos are most influential in changing misperceptions (and encouraging spill-over to misperceptions not covered in the videos), the extent to which the videos result in long-term changes in behavior and how we can encourage our key target groups to watch and engage with this important misperception debunking content.

## Supplementary Material

A Contributorship Form detailing each author’s specific involvement with this content, as well as any supplementary data, are available online at https://academic.oup.com/ntr.

ntab104_suppl_Supplementary_Materials

ntab104_suppl_Supplementary_Taxonomy_form

## Funding

This work was supported by funding awarded to O.M.M. by the Economic and Social Research Council (ESRC; ES/R003424/1).

## Declaration of Interests

*All authors declare no conflicts of interest*.

## Data Availability

Data are available at the University of Bristol data repository, data.bris, at https://data.bris.ac.uk/data/dataset/8j9tx4rrrfu822xmuluahaunb; DOI: 10.5523/bris.8j9tx4rrrfu822xmuluahaunb.
