# Supplementary material for: Tackling Smoker Misperceptions About E-cigarettes Using Expert Videos
Source: Nicotine Tob Res. 2021 May 20;23(11):1848–54. doi: 10.1093/ntr/ntab104 (PMC8496477; doi:10.1093/ntr/ntab104)
Supplement: ntab104_suppl_Supplementary_Materials [file ntab104_suppl_supplementary_materials.docx]

Supplementary Materials

Tackling Smoker Misperceptions about E-cigarettes using Expert Videos

Madeleine Svenson^1,2^, James Green^2^ and Olivia M. Maynard^2^

**
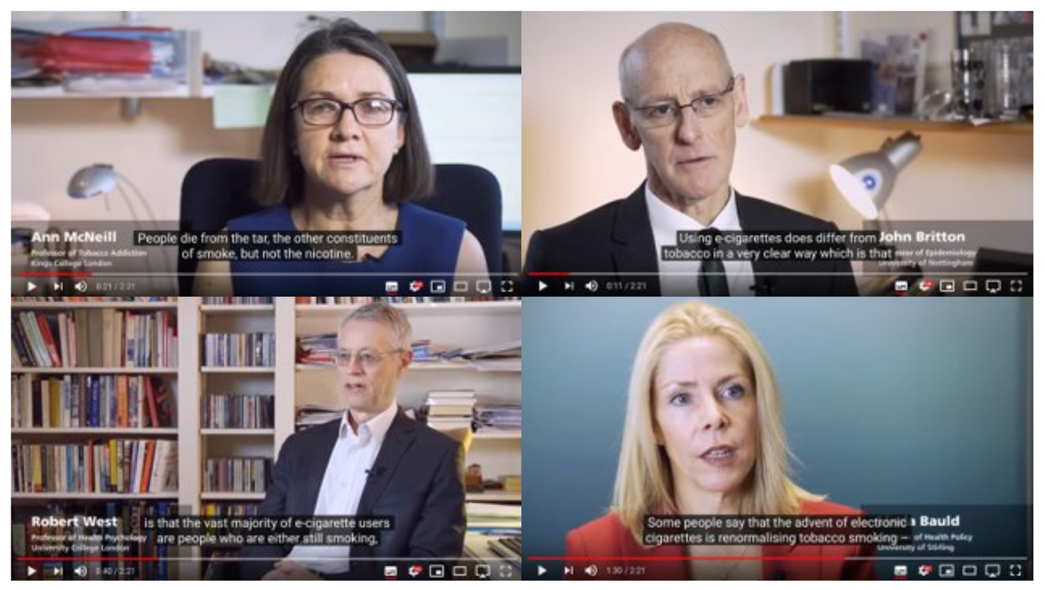

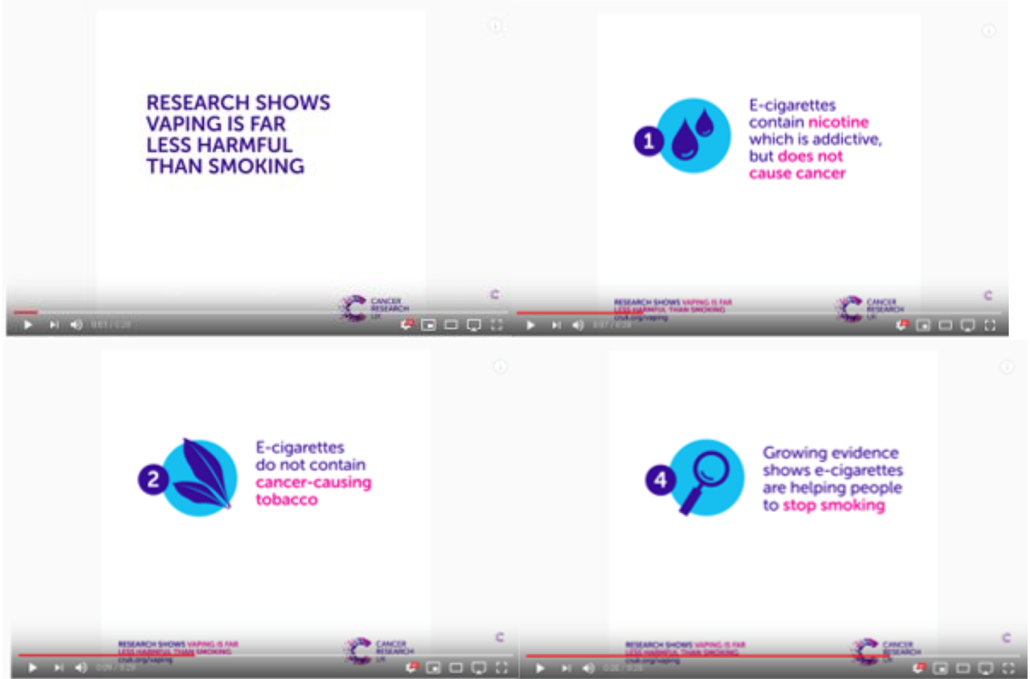
Supplementary Figure 1:** *Example stills from CRUK and expert videos (consent to publish images of those pictured has been obtained).*

**Expert Video transcript:**

Text: E-cigarette safety: The facts explained

Speech:

**John Britton** Professor of Epidemiology University of Nottingham: Using e-cigarettes does differ from tobacco in a very clear way which is that it doesn’t involve inhaling tobacco smoke, and as the constituents of smoke are the things the kill smokers that has to be a good thing.

**Ann McNeill** Professor of Tobacco Addiction King College London: People die from the tar, the other constituents of smoke, but not the nicotine. So the nicotine isn’t the harmful component and electronic cigarettes allow smokers to get the nicotine without all the other harmful stuff that comes along in cigarette smoke.

**Robert West** Professor of Health Psychology University College London: The first thing to note when considering the safety of e-cigarettes is that the vast majority of e-cigarette users are people who are either still smoking, and using them to cut down on the amount they smoke or have used them to stop smoking.

**John Britton**: If we try to put a figure on the relative risk of electronic cigarettes compared with smoking, my view us that it is going to be well under 5% of the risk possible slightly more for cardiovascular disease but substantially less from chronic obstructive pulmonary disease and lung cancer.

**Robert West**: When you look at the concentrations of and the nature of the toxins in cigarette smoke, and then you compare that with e-cigarettes you see that really there’s no comparison. Most of the toxins in cigarette smoke aren’t present at all in e-cigarettes vapour those that are present are in concentration that are a hundred times less or more.

**Linda Bauld**Professor of Health Policy the University of Stirling: Some people say the advent of electronic cigarettes is renormalizing tobacco smoking – so we see people using these devices, and its making smoking more normal again. Interestingly we are just not seeing that in the data we have.

**John Britton**: All that it does is normalise electronic cigarette use. And if we could normalise electronic cigarette use for the nearly 9 million people in the United Kingdom who are still addicted to tobacco that could only be a good thing.

**Robert West**: I think it’s really important that people be reassured that e-cigarettes, whatever you might see in the press (electronic cigarettes) are considerably safer than smoking and the evidence is pretty good that they can be effective in helping people to stop (smoking).

**Supplementary Table 1.** *Participant characteristics – continued*

| **Participant Characteristic** | **All participants (n = 382)** | **Control (n=132)** | **CRUK**  **(n= 121)** | **Expert (n=129)** |
| --- | --- | --- | --- | --- |
| **Education** |  |  |  |  |
| Higher Education or professional/vocational equivalents | 134 (35%) | 47 (36%) | 42 (35%) | 45 (35%) |
| A-levels* or vocational level 3 or equivalents | 105 (28%) | 38 (29%) | 32 (26%) | 35 (27%) |
| GCSE/O-Level* grade A*-C, or vocational level 2 or equivalents | 98 (26%) | 34 (26%) | 26 (22%) | 38 30%) |
| GCSE/O Level* grace C or below, or qualification at level 1 or below | 28 (7%) | 7 (5%) | 13 (11%) | 8 (6%) |
| Other qualifications level unknown | 9 (2%) | 3 (2%) | 6 (5%) | 0 (0%) |
| No qualifications | 8 (2%) | 3 (2%) | 2 (2%) | 3 (2%) |
|  |  |  |  |  |
| **Student Status** |  |  |  |  |
| Students | 20 (5%) | 7 (5%) | 6 (5%) | 7 (5%) |
| Undergraduate | 16 (4%) | 4 (3%) | 6 (5%) | 6 (5%) |
| Postgraduate | 4 (1%) | 3 (2%) | 0 (0%) | 1 (1%) |
|  |  |  |  |  |
| **Profession of chief earner** |  |  |  |  |
| Higher managerial/ professional/ administrative (e.g.  Established doctor, Solicitor, Board Director in a large organisation (200+ employees, top level civil servant/public service employee) | 16 (4%) | 7 (5%) | 4 (3%) | 5 (4%) |
| Intermediate managerial/ professional/ administrative (e.g.  Newly qualified (under 3 years) doctor, Solicitor, Board director  small organisation, middle manager in large organisation, principal officer in civil service/local government) | 71 (19%) | 26 (20%) | 23 (19%) | 22 (17%) |
| Supervisory or clerical/ junior managerial/ professional/  administrative (e.g. Office worker, Student Doctor, Foreman with  25+ employees, salesperson, etc.)18 | 106 (28%) | 34 (26%) | 41 (34%) | 31 (24%) |
| Student | 7 (2%) | 5 (26%) | 2 (2%) | 0 (0%) |
| Skilled manual worker (e.g. Skilled Bricklayer, Carpenter,  Plumber, Painter, Bus/ Ambulance Driver, HGV driver, AA  patrolman, pub/bar worker, etc.) | 65 (17%) | 18 (14%) | 20 (17%) | 27 (21%) |
| Semi or unskilled manual work (e.g. Manual workers, all apprentices to be skilled trades, caretaker, parker keeper, non-HGV driver, shop assistant) | 59 (15%) | 20 (15%) | 16 (13%) | 23 (18%) |
| Casual worker – not in permanent employment | 2 (1%) | 2 (2%) | 0 (0%) | 0 (0%) |
| Housewife/ Homemaker | 7 (2%) | 3 (2%) | 1 (1%) | 3 (2%) |
| Retired and living on state pension | 9 (2%) | 5 (4%) | 2 (2%) | 2 (2%) |
| Unemployed or not working due to long-term sickness | 36 (9%) | 10 (8%) | 11 (9%) | 15 (12%) |
| Full-time carer of another household member | 4 (1%) | 2 (2%) | 1 (1%) | 1 (1%) |
|  |  |  |  |  |
| **Ethnicity** |  |  |  |  |
| English/Welsh/Scottish/Northern Irish/British | 330 (86%) | 114 (86%) | 103 (85%) | 113 (88%) |
| White other | 26 (7%) | 9 (7%) | 8 (7%) | 9 (7%) |
| White and Black Caribbean | 5 (1%) | 1 (0.3%) | 3 (0.8%) | 1 (0.3%) |
| White Asian | 4 (1%) | 1 (0.3%) | 1 (0.3%) | 2 (0.5%) |
| Caribbean | 4 (1%) | 1 (0.3%) | 1 (0.3%) | 2 (0.5%) |
| Indian | 3 (0.8%) | 2 (0.5%) | 1 (0.3%) | 0 (0%) |
| Pakistani | 3 (0.8%) | 1 (0.3%) | 1 (0.3%) | 1 (0.3%) |
| Other Asian background | 2 (0.5%) | 1 (0.3%) | 1 (0.3%) | 0 (0%) |
| African | 2 (0.5%) | 1 (0.3%) | 0 (0%) | 1 (0.3%) |
| Irish | 1 (0.3%) | 0 (0%) | 1 (0.3%) | 0 (0%) |
| Chinese | 1 (0.3%) | 0 (0%) | 1 (0.3%) | 0 (0%) |
| Any other ethnic group | 1 (0.3%) | 1 (0.3%) | 0 (0%) | 0 (0%) |

** A-Levels are UK school exams usually taken age 16-18. GCSEs/O-levels are UK school exams usually taken age 14-16.*

**Supplementary Table 2.** *Games Howell post-hoc contrasts comparing mean harm perception scores between conditions*

|  | Control compared to expert  (control – expert) | | | | Control compared to CRUK  (control – CRUK) | | | | CRUK compared to expert  (CRUK – expert) | | | |
| --- | --- | --- | --- | --- | --- | --- | --- | --- | --- | --- | --- | --- |
|  | MD | SE | p | d | MD | SE | p | d | MD | SE | p | d |
| **General Harm Perception Measures** | | | | | | | | | | | | |
| E-cigarettes are a helpful tool for people who want to quit smoking | 0.9 | 0.1 | <0.001 | 0.77 | 0.5 | 0.2 | 0.008 | 0.38 | 0.5 | 0.2 | 0.008 | 0.38 |
| There is convincing scientific evidence that e-cigarettes are safe | 2.0 | 0.1 | <0.001 | 1.73 | 1.2 | 0.2 | <0.001 | 0.88 | 0.8 | 0.2 | <0.001 | 0.61 |
| There is convincing scientific evidence that e-cigarettes are safer than smoking | 1.5 | 0.2 | <0.001 | 1.19 | 0.7 | 0.2 | 0.002 | 0.45 | 0.9 | 0.2 | <0.001 | 0.61 |
| I know enough about e-cigarettes to have formed accurate opinions | 0.7 | 0.2 | 0.002 | 0.43 | 0.4 | 0.2 | 0.089 | 0.27 | 0.3 | 0.2 | 0.420 | 0.16 |
| E-cigarettes are less harmful than combustible cigarettes | 1.5 | 0.2 | <0.001 | 1.17 | 0.7 | 0.2 | <0.001 | 0.50 | 0.7 | 0.2 | <0.001 | 0.59 |
| E-cigarettes are harmful* | -1.2 | 0.2 | <0.001 | -0.94 | -1.0 | 0.2 | <0.001 | -0.78 | -0.3 | 0.2 | 0.353 | -0.18 |
| **Specific Harm Perception Measures** | | | | | | | | | | | | |
| E-cigarettes often contain chemicals that are harmful to the user’s health* | -1.6 | 0.2 | <0.001 | -1.21 | -1.1 | 0.2 | <0.001 | -0.84 | -0.5 | 0.2 | 0.044 | -0.32 |
| The health risks of smoking come from the nicotine in combustible cigarettes* | -1.7 | 0.2 | <0.001 | -0.96 | -0.5 | 0.2 | 0.134 | -0.25 | -1.2 | 0.2 | <0.001 | -0.65 |
| E-cigarettes often contain tar* | -1.1 | 0.2 | <0.001 | -0.93 | -0.5 | 0.2 | 0.022 | -0.35 | -0.6 | 0.2 | 0.002 | -0.47 |
| There is a high risk of harmful accidents when using e-cigarettes* | -1.5 | 0.2 | <0.001 | -1.08 | -0.7 | 0.2 | <0.001 | -0.53 | -0.8 | 0.2 | <0.001 | -0.55 |
| Second-hand e-cigarette vapour can expose others to harm* | -1.2 | 0.2 | <0.001 | -0.86 | -1.3 | 0.2 | <0.001 | -0.99 | 0.1 | 0.2 | 0.892 | 0.06 |
| E-cigarettes normalise smoking, making more young people take up smoking* | -1.0 | 0.2 | <0.001 | -0.61 | -0.1 | 0.2 | 0.800 | -0.08 | -0.9 | 0.2 | <0.001 | -0.54 |
| The health risks of smoking come from the tar in combustible cigarettes | 0.7 | 0.1 | <0.001 | 0.61 | 0.1 | 0.2 | 0.789 | 0.09 | 0.6 | 0.1 | <0.001 | 0.52 |

Mean difference (MD) scores are calculated by subtracting the mean score from one condition (e.g. expert) from another (e.g. control). The asterisks denote where negative mean difference scores indicate a more favourable opinions of e-cigarettes by the latter group in the comparison (rather than the reverse for other items). Standard Error (SE).
